# Long-term effect of apomorphine infusion in advanced Parkinson’s disease: a real-life study

**DOI:** 10.1038/s41531-021-00194-7

**Published:** 2021-06-11

**Authors:** Bruna Meira, Bertrand Degos, Elise Corsetti, Mohamed Doulazmi, Emeline Berthelot, Clara Virbel-Fleischman, Pauline Dodet, Aurélie Méneret, Louise-Laure Mariani, Cécile Delorme, Florence Cormier-Dequaire, David Bendetowicz, Nicolas Villain, Clément Tarrano, Lise Mantisi, Hélène Letrillart, Céline Louapre, Eavan McGovern, Yulia Worbe, David Grabli, Marie Vidailhet, Elodie Hainque, Emmanuel Roze

**Affiliations:** 1 Neurology Department, Centro Hospitalar Lisboa Ocidental, Lisbon, Portugal; 2 Neurology Department, Avicenne Hospital, AP-HP, Sorbonne Paris Nord University, Bobigny, France; 3 Neurology Department, Pitié-Salpêtrière Hospital, AP-HP, Paris, France; 4 Adaptation Biologique et Vieillissement, Institut de Biologie Paris Seine, Sorbonne University, CNRS, Paris, France; 5 Neurology Department, Hôpital Zobda Quitman, Fort-de-France, French West Indies France; 6 Air Liquide SA, Explor Center (Healthcare), Fort-de-France, Paris France; 7 Paris Brain Institute, Sorbonne University, UMR S 1127, Inserm U 1127, CNRS UMR 7225, Paris, France; 8 Sleep Disorders (Department “R3S”), Pitié-Salpêtrière Hospital, Paris, France; 9 Sorbonne Université, GRC no 21, Alzheimer Precision Medicine, Paris, France; 10 Neurophysiology Department, Saint Antoine Hospital, AP-HP, Paris, France

**Keywords:** Parkinson's disease, Parkinson's disease, Quality of life

## Abstract

Long-term effects of continuous subcutaneous apomorphine infusion (CSAI) on health-related quality of life (HRQoL) and predictors of CSAI discontinuation are poorly known. Data from consecutive advanced Parkinson’s disease patients treated in routine care were retrospectively collected over 24 months after CSAI initiation, with a focus on the 39-item Parkinson’s disease questionnaire (PDQ-39). We determined predictors of CSAI discontinuation and HRQoL improvement using multiple regression analysis. Of the 110 subjects evaluated over a 2-year period, 35% discontinued CSAI. Of those who continued treatment, HRQoL remained stable with a sustained reduction in motor fluctuations. The observed effect on dyskinesias was mild and transient. Of note, patients with preexisting impulse control disorders showed an overall good tolerability. PDQ-39 was the only baseline predictor of HRQoL improvement after 2 years of treatment. The presence of dyskinesias, poorer psychological status, shorter disease duration, male sex, and worse OFF state were predictors of discontinuation. Best candidates for CSAI are patients with: (i) poor baseline HRQoL and (ii) marked motor fluctuations.

## Introduction

Oral dopaminergic therapy is effective in early Parkinson’s disease (PD)^1^. With disease progression, motor complications (fluctuations and dyskinesia) emerge. Attempts to control motor fluctuations with oral medication tend to worsen dyskinesias^2,3^.

Motor fluctuations can be managed with continuous dopaminergic drug delivery using either continuous subcutaneous apomorphine infusion (CSAI) or levodopa–carbidopa intestinal gel (LCIG)^4,5^. Apomorphine is a dopamine agonist with affinity for D1 and D2 receptors^6^. Several uncontrolled studies have highlighted the efficacy of apomorphine in managing motor complications^7–9^ and nonmotor symptoms^10–13^ in patients with advanced PD. More recently, the efficacy, tolerability, and safety of CSAI were demonstrated in a short-term, large, prospective, randomized, placebo-controlled study^14^.

Despite low internal validity, real-life studies represent an interesting option to study long-term effects of a treatment and the clinical relevance of randomized controlled trials (RCT) findings. Health-related quality of life (HRQoL) is a meaningful outcome measure as it captures patient-centered issues such as daily life functioning and medication-related adverse effects.

Positive short-term effects of CSAI on HRQoL have been observed using Parkinson disease questionnaire 39 (PDQ-39) or its shorter version in real-life studies^11–13,15–17^. However, little is known about the long-term effects of CSAI on HRQoL, as most studies involved a 6-month follow-up period^12,13,15,16^. Only two studies had a longer follow-up period (12.5 (11.5) and 27.9 (24.9) months, respectively) but were limited by small cohort sizes and inconsistent findings^9,15^. Furthermore, predictors of CSAI discontinuation were not addressed and remain largely unknown^18,19^.

We conducted a retrospective analysis of a large series of consecutive advanced PD patients treated with CSAI over a 6-year period at the Salpêtrière Hospital in Paris and described their 2-year follow-up. We focused on the evolution of HRQoL, motor fluctuations, and dyskinesia 2 years after CSAI onset and attempted to identify predictors of CSAI discontinuation and HRQoL improvement.

## Results

### Cohort’s description

**Fig. 1:**
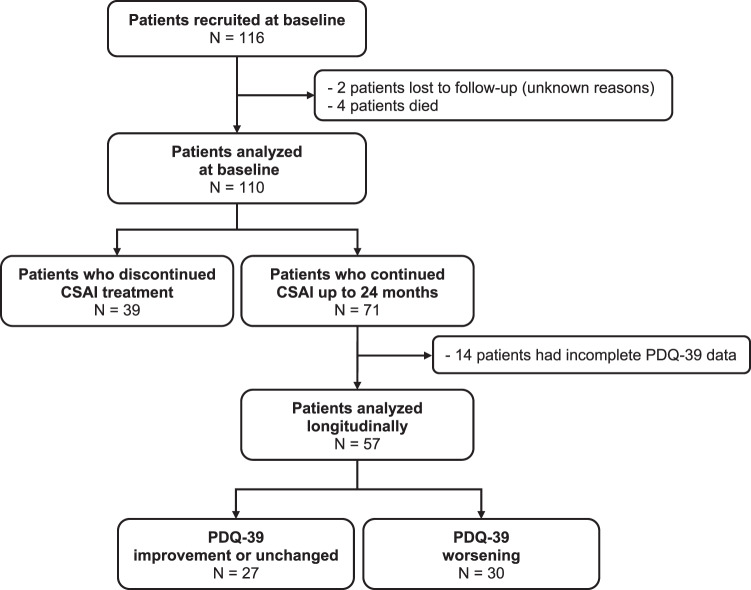
Study flow chart. CSAI continuous subcutaneous apomorphine infusion, PDQ-39 Parkinson disease questionnaire 39.

One-hundred and sixteen PD patients started CSAI treatment. Six patients were subsequently excluded from the analysis: four died during the study period (the cause of death was unrelated to CSAI in two and unknown in two) and two were lost to follow-up. A total of 110 patients were analyzed (Fig. 1): 55 male patients, mean age 62.9 (9.6) years, mean age at PD onset 50.9 (8.7) years, 47% with an akinetic-rigid motor subtype, mean PD duration 12 (6.1) years, median Hoehn and Yahr scale (H&Y) score OFF-state 3 and ON-state 2, mean motor fluctuations duration 4.5 (3.6) years, mean ON unified Parkinson’s disease rating scale part II (UPDRS-III) 19 (13), and mean UPDRS-IV 8.6 (3.5). Thirty-eight patients had a past history (Supplementary Fig. 1) of impulse control disorders (ICDs) and 55 of depression (mean Ardouin scale of behavior in Parkinson’s disease (ASPBD) 7.4 (4.3)). Of the 110 patients analyzed, 71 continued treatment up to the 24-month assessment and 39 patients discontinued the CSAI within the 2 years of follow-up. Table 1 shows the comparison of baseline characteristics between both groups and Supplementary Information reports the description of the patients aged over 80 years.

**Table 1:** Baseline characteristics.

	Continued CSAI therapy (*n* = 71)	Discontinued CSAI therapy (*n* = 39)	*p* value
Demographics			
Male	32 (45)	23 (59)	0.163^a^
Age, years	63 (9.7)	62 (9.6)	0.342^b^
PD characteristics			
Age at PD onset, years	51 (9)	51 (8.2)	0.938^b^
Disease duration, years	13 (5.9)	11 (6.4)	0.0607^c^
Motor fluctuations duration, years	5.1 (3.8)	3.5 (3)	0.0184^c^
Dyskinesias, yes	42 (60)	27 (79)	0.0494^a^
Motor status			
UPDRS-II, ON	8.6 (7.6)	9.1 (7.7)	0.644^c^
UPDRS-II, OFF	22 (9.2)	21 (8.5)	0.505^c^
UPDRS-III, ON	19 (13)	17 (12)	0.507^c^
UPDRS-IV total	8.1 (3.5)	9.3 (3.6)	0.101^b^
Dysk UPDRS	3.6 (2.9)	4.2 (2.7)	0.172^c^
Fluc UPDRS	3.5 (1.5)	3.9 (1.3)	0.186^c^
Hoehn and Yahr, OFF	3.1 (1.1)	3.2 (1.1)	0.791^c^
Schwab and England, OFF	55 (24)	47 (21)	0.0594^c^
Cognitive status			
MMSE	27 (3)	27 (3.5)	0.346^c^
FAB	16 (2.6)	15 (2.9)	0.71^c^
UPDRS-I	2.9 (2)	3.2 (2.1)	0.681^c^
ASBPD total	8.1 (4.6)	7.1 (4.3)	0.265^c^
ASBPD-1	2.9 (2.2)	3.9 (3)	0.115^c^
ASBPD-2	0.50 (0.8)	0.49 (0.8)	0.754^c^
ASBPD-3	1.3 (1.3)	1.4 (1.3)	0.462^c^
ASBPD-4	2.4 (2.5)	2.2 (1.1)	0.733^c^
PDQ-39 total score	42.6 (12.5)	43.1 (14.4)	0.870^b^
PD treatment history			
Total LEDD, mg	1299 (461)	1378 (665)	0.956^c^
Dopamine agonists, LEDD, mg	130 (132)	150 (229)	0.848^c^
Amantadine	26 (37)	11 (28)	0.372^a^
Clozapine	7 (10)	2 (5)	0.695^a^
Antidepressants	29 (41)	17 (45)	0.451^a^

Values are mean (SD) or *n* (%).

*CSAI* continuous subcutaneous apomorphine infusion, *PD* Parkinson disease, *UPDRS* unified Parkinson’s disease rating scale, *UPDRS*-I UPDRS part I, mentation, behavior and mood, *UPDRS*-II UPDRS part II, activities of daily living, *UPDRS*-III UPDRS part III, motor examination, *UPDRS*-IV UPDRS part IV, complications of therapy, *Dysk* UPDRS items 32–35 of UPDRS-IV, *Fluc* UPDRS items 36–39 of UPDRS-IV, *MMSE* mini mental state examination, *FAB* frontal assessment battery, *ASBPD* Ardouin scale of behavior in Parkinson’s disease, *ASBPD*-1 ASBPD part 1, general psychological state, *ASBPD*-2 ASBPD part 2, overall functioning in apathetic mode, *ASBPD*-3 ASBPD part 3, nonmotor fluctuations, *ASBPD*-4 ASBPD part 4, hyperdopaminergic behaviors, *PDQ*-39 Parkinson disease questionnaire 39, *LEDD* levodopa equivalent daily dose.

^a^Pearson’s *χ*^2^ test.

^b^Student’s *t* test.

^c^Mann–Whitney test.

### Quality of life and treatment satisfaction assessment

**Fig. 2:**
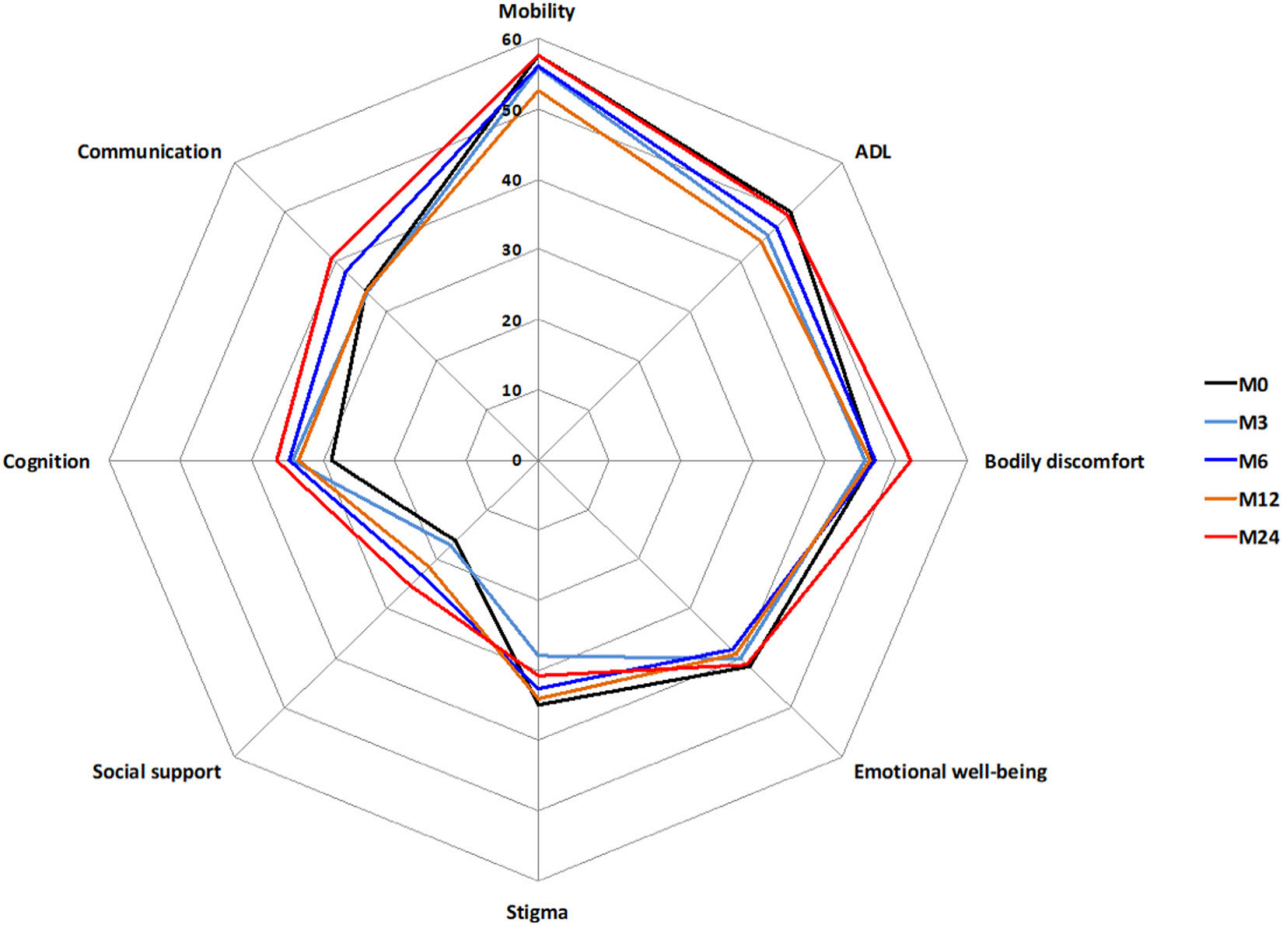
Comparison of HRQoL measured by PDQ-39. The evolution of each dimension of PDQ-39 at baseline (M0) and at each follow-up visit (M3, M6, M12, M24).

Of the 71 patients who continued treatment for 24 months, 14 patients were excluded from the analysis due to incomplete baseline and/or 24-month PDQ-39 assessment data (Fig. 1). No difference in baseline characteristics was observed between patients excluded and patients analyzed. Of the 57 remaining patients, total PDQ-39 score (Table 2 and Fig. 2) was unchanged from M0 to M24 (*p* = 0.054). The trend toward significance seen on longitudinal analysis was not observed on post-hoc analysis (M24 compared to M0, *p* > 0.23). Stigma dimension showed the most significant improvement (34.96 at M0 vs 28.84 at M3 (*p* = 0.02) and 30.83 at M24 (*p* = 0.018)). A significant improvement in mobility dimension was observed at M12 (57.68 at M0 vs 52.73 at M12, *p* = 0.028) but was no longer present at M24. Conversely, social support showed the largest significant deterioration (+55.8%) at M24. The communication dimension was also significantly altered at M24 (+19.6%). The remaining dimensions (activities of daily living, body discomfort, emotional well-being, cognitions) were unchanged.

**Table 2:** Assessment of patient’s quality of life measured by PDQ-39 (*n* = 57).

Dimension	M0	M3	M6	M12	M24	Friedman test *p* value	Relative change M0–M24
Physical score	53.5 (18.1)	51.3 (18.3)	51.9 (17.6)	48.9 (17.5)^a^	54.1 (17.0)	0.013	+1.2%
Mobility	57.7 (22.7)	56.0 (22.5)	56.2 (20.6)	52.7 (19.9)^a^	57.6 (18.8)	0.036	−0.09%
ADL	49.9 (20.3)	45.2 (20.0)	47.0 (20.6)	44.0 (20.4)	49.3 (22.7)	0.134	−1%
Bodily discomfort	46.9 (20.6)	45.7 (19.7)	47.1 (21.2)	46.4 (20.1)	52.1 (19.6)	0.297	+11%
Psychological score	32.8 (11.5)	32.0 (13.4)	34.3 (12.6)	33.3 (13.6)	35.6 (12.4)	0.366	+8%
EMO	41.7 (19.0)	40.1 (17.6)	38.3 (16.2)	39.0 (17.5)	41.2 (15.8)	0.871	−1.4%
Stigma	35.0 (20.3)	28.0 (21.9)^a^	32.6 (25.8)	34.1 (23.1)^a^	30.8 (22.9)^a^	0.027	−11.8%
Social support	16.2 (17.8)	17.3 (21.2)	23.1 (23.7)	21.5 (21.6)	25.3 (21.9)^a^	0.015	+55.8%
Cognition	28.8 (17.7)	34.2 (22.0)	34.8 (17.8)	33.5 (18.3)	36.4 (18.4)	0.387	+26%
Communication	34.1 (19.5)	33.8 (22.2)	38.0 (22.2)	33.9 (19.2)	40.8 (19.6)^a^	0.018	+19.6%
PDQ-39 total	42.3 (12.3)	40.8 (13.7)	42.9 (14.8)	40.3 (14.0)	44.3 (13.4)	0.054	+4.6%

Changes for each dimension, physical or psychological subscores, and PDQ-39 total score. Data are mean (SD). The PDQ-39 range is 0–100; the higher the score, the worse the self-reported quality of life; negative change = improvement. Missing values in PDQ-39 domain scores were imputed using the nearest available observations. Related samples Friedman’s two-way analysis of variance by ranks test followed by Wilcoxon tests with a Bonferroni Correction applied.

*PDQ-39* 39-item Parkinson’s disease questionnaire, AD activities of daily living, *EMO* emotional well-being.

^a^*p* value < 0.05 compared to M0. Since there was a strong trend toward significance for PDQ-39 total (*p* = 0.054), we checked the score evolution compared to M0 by paired comparisons and found that all the *p* values were >0.23; relative change = (mean Testfollow-up − mean Testbaseline)/Testbaseline × 100.

Of the 57 patients, PDQ-39 improved from M0 to M24 in 26 patients, remained unchanged in one patient, and deteriorated for the remaining (30 patients).

**Fig. 3:**
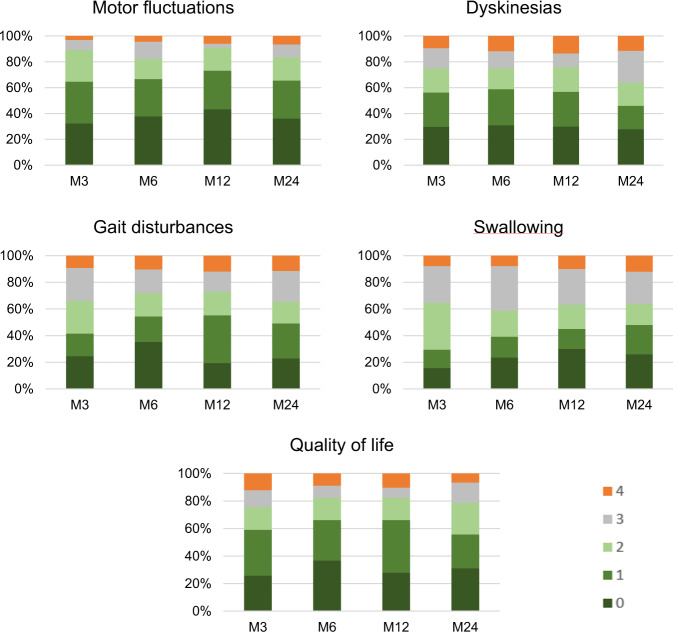
Satisfaction self-assessment questionnaire. Histograms illustrating the proportion of patients for each score on the five items of the treatment satisfaction self-questionnaire. Motor fluctuations was the item with the largest percentage of patients stating improvement (83–91%). Quality of life was improved in 76–82% of patients and dyskinesias in 64–76%. Gait and swallowing disturbances showed a more modest percentage (60–73%). 0 = very much improved, 1 = moderately improved, 2 = little improved, 3 = not improved, and 4 = deteriorated.

The satisfaction’s self-questionnaire (Fig. 3) reported an improvement (score between 0 and 2) on five items in more than 65% of the patients. Perceived improvement was maximal at M12 (motor fluctuations were improved in 91% of the patients, dyskinesias in 76% and quality of life in 82%). At M24, motor fluctuations were reported to be improved in 84% of the patients, dyskinesias in 64% and quality of life in 79%. The device satisfaction’s self-questionnaire showed a good general satisfaction (5.9 (2.3)), a moderate device’s comfort (5.3 (2.4)), and it was evaluated as not particularly painful (2.3 (2.6)).

### Motor and nonmotor symptoms assessments

Table 3 shows the motor and nonmotor assessment evolution with CSAI treatment. Fluc UPDRS subscore improved by 32.4% at M24 (*p* < 0.001). Dysk UPDRS and UPDRS-III scores remained stable over the 24 months (*p* = 0.394 and *p* = 0.07, respectively). ON UPDRS-II score showed a significant deterioration at M24 (*p* < 0.001). However, such deterioration was not found for OFF UPDRS-II score (*p* = 0.629).

**Table 3:** Changes in motor, nonmotor, and cognitive dimensions with CSAI treatment (*n* = 57).

	M0	M3	M6	M12	M24	Friedman test *p* value
Motor status						
UPDRS-II,ON	7.9 (6.6)	8.3 (5.8)	8.6 (7.2)	9.1 (6.8)	12.0 (8.6)^c^	0.002
UPDRS-II, OFF	22.2 (9.1)	20.8 (8.0)	20.4 (7.7)	20.5 (7.8)	21.8 (9.7)	0.629
UPDRS-III, ON	18.5 (11.1)	16.8 (13.2)	18.7 (13.4)	18.8 (13.6)	22.3 (14.1)	0.07
UPDRS-IV	8.1 (3.0)	7.2 (3.6)	7.2 (3.6)	7.4 (3.7)	8.0 (3.9)	0.960
Dysk UPDRS	3.4 (2.7)	3.0 (2.5)	3.4 (3.0)	3.4 (2.7)	3.9 (2.7)	0.394
Fluc UPDRS	3.7 (1.4)	2.9 (1.4)^b^	2.7 (1.5)^a^	2.6 (1.6)^b^	2.5 (1.6)^c^	<0.001
H&Y, OFF	3.0 (1.1)	3.1 (1.2)	3.0 (1.1)	3.0 (1.0)	3.3 (1.1)	0.264
S&E, OFF	60 (20)	60 (20)	60 (20)	60 (20)	50 (30)	0.422
Cognitive/nonmotor status						
UPDRS-I	3.0 (1.8)	2.6 (2.0)	2.5 (2.0)	3.0 (2.6)	4.0 (2.7)^b^	<0.001
MMSE	27.4 (3.1)	26.7 (3.5)	27.1 (3.4)	27.4 (3.8)	26.5 (4.1)	0.041
FAB	15.7 (2.6)	15.4 (2.9)	15.9 (2.4)	15.7 (2.5)	14.9 (3.2)^b^	0.003
ASBPD total score	6.6 (4.4)	6.4 (4.5)	6.5 (4.1)	6.4 (4.1)	7.0 (3.7)	0.319
ASBPD-1	2.6 (2.1)	3.0 (2.6)	3.0 (2.5)	3.1 (2.3)	3.5 (2.5)	0.106
ASBPD-2	0.5 (0.7)	0.4 (0.7)	0.2 (0.5)	0.2 (0.5)	0.6 (0.9)	0.019
ASBPD-3	1.4 (1.3)	0.9 (1.0)	1.0 (1.0)	1.0 (1.1)	0.8 (0.9)	0.06
ASBPD-4	2.3 (2.6)	2.1 (2.1)	2.1 (1.8)	2.2 (2.3)	2.2 (1.8)	0.778

Values presented are mean (SD). Post-hoc analysis with Wilcoxon signed-rank test was conducted with a Bonferroni correction applied.

*CSAI* continuous subcutaneous apomorphine infusion, *PD* Parkinson disease, *UPDRS* unified Parkinson’s disease rating scale, *UPDRS*-I UPDRS part I, mentation, behavior and mood, *UPDRS*-II UPDRS part II, activities of daily living, *UPDRS*-III UPDRS part III, motor examination, *UPDRS*-IV UPDRS part IV, complications of therapy, *Dysk* UPDRS items 32–35 of UPDRS-IV, *Fluc* UPDRS items 36–39 of UPDRS-IV, *H&Y* Hoehn and Yahr scale, *S&E* Schwab and England activities of daily living scale, *MMSE* mini mental state examination score, *FAB* frontal assessment battery, *ASBPD* Ardouin scale of behavior in Parkinson’s disease, *ASBPD*-1 ASBPD part 1, general psychological state, *ASBPD*-2 ASBPD part 2, overall functioning in apathetic mode, *ASBPD*-3 ASBPD part 3, nonmotor fluctuations, ASBPD-4 ASBPD part 4, hyperdopaminergic behaviors.

^a^*p* value < 0.05 compared to M0 by Wilcoxon signed-rank test.

^b^*p* value < 0.01 compared to M0 by Wilcoxon signed-rank test.

^c^*p* value < 0.001 compared to M0 by Wilcoxon signed-rank test.

At M24, we found a significant deterioration on UPDRS-I (*p* < 0.001), mini mental state examination (MMSE) (*p* = 0.041), and frontal assessment battery (FAB) scale (*p* = 0.003). Conversely, total Ardouin scale of behavior in Parkinson’s disease (ASBPD) and ASBPD-4 did not change significantly.

### Treatment adjustments

Treatment adjustments were analyzed in patients who continued CSAI over the 2-year period (Table 4). Six patients had already been treated by DBS before CSAI onset. Most patients (71%) were treated with 24-h continuous infusion from CSAI initiation and 15 patients (21%) received CSAI treatment only during daytime throughout the study. Only two patients achieved sustained withdrawal of oral antiparkinsonian medications (one of them had concomitant DBS). Six patients achieved withdrawal at some point but resumed levodopa during the study follow-up. With CSAI introduction, 79, 59, and 57% of patients receiving dopamine agonists, COMT inhibitors, and MAO-B inhibitors at M0 discontinued these treatments, respectively. Reasons to continue dopamine agonist were: nocturnal/early morning OFFs and restless leg syndrome. The percentage of patients receiving amantadine did not differ from M0 to M24 (38–35%). Twenty-one patients received clozapine at M24 (31%). Of those, 7/21 (10%) received clozapine before CSAI initiation and continued to receive it. Reasons to introduce clozapine in the remaining 14/21 patients were: hallucinations (5), ICD (6), severe mood disorders (1), dyskinesia (1), and sleep disorder (1).

**Table 4:** Treatment adjustments in patients who continued CSAI over the 2-year period (*n* = 71).

	M0	M24^c^
Total LEDD, mg/day	1304.4 (461.8)^a^	1725.4 (671.5)
Oral LEDD, mg/day	1298.6 (461.2)^a^	754.0 (465.5)
Daily L-dopa dose, mg/day	998.5 (359.4)^a^	670.2 (435.8)
Dopamine agonist, *n* (%)	48 (70%)^a^	10 (15%)
Dopamine agonist LEDD, mg/day	186.4 (120.1)^a,d^	94 (61.4)^d^
Daily CSAI total dose, mg/day	78.9 (38.9)^b^	94.8 (51.8)
Daytime CSAI dose, mg/h	4.7 (2.1)^b^	4.9 (2.4)
Daytime infusion duration, h/day	13.1 (2.8)^b^	14.2 (3.4)
Nighttime CSAI dose, mg/h	1.5 (1.7)^b^	2.5 (2.1)
Night infusion duration, h/day	5.6 (5.4)^b^	6.8 (4.1)
COMT inhibitors, *n* (%)	34 (49%)^a^	14 (21%)
COMT inhibitors-LEDD, mg/day	340.1 (117.3)^a,d^	274.4 (122.4)^d^
MAO inhibitors, *n* (%)	14 (20%)^a^	6 (9%)
MAO inhibitors-LEDD, mg/day	101.8 (34.6)^a,d^	100 (0)^d^
Amantadine, *n* (%)	26 (38%)^a^	23 (35%)
Amantadine, mg/day	203.8 (72.0)^a,d^	217.4 (77.8)^d^

Treatment information are present in *n* (%) = number of patients receiving dopamine agonists, COMT inhibitors, MAO-B inhibitors, and amantadine and treatment doses are presented as mean (SD).

^a^Values at admission at M0.

^b^Values at discharge at M0.

^c^Values at admission at M24.

^d^Mean doses values calculated for the group of patients receiving the treatment.

### Adverse effects of CSAI

Prevalence of adverse effects related to CSAI was analyzed in patients who continued CSAI over the 2-year period (Table 5). The most common adverse event was injection site skin nodules; its occurrence was stable throughout the study (53 to 57% of patients). These nodules were not particularly painful and, on average, patients attributed 1 on the numerical pain rating scale. Digestive symptoms (nausea or vomiting) and orthostatic hypotension were particularly common during the first week of treatment (33.3% and 27.5% respectively). During follow-up, neuropsychiatric adverse events were reported in 20-30% of patients. Mild to moderate hallucinations were the most frequent and the rate of affected patients increased gradually during the 24 months of follow-up (18.6% of patients at M3 and 37.5% at M24). Mild to moderate ICDs were observed over time (29.3% at M3 and 18.9% at M24) such as compulsive eating, compulsive shopping, hypersexuality, pathological gambling, punding, hobbyism, and hyperactivity. More marked ICDs were noted in seven patients (punding, compulsive eating, compulsive shopping and pathological gambling). Among the 38 patients with a previous history of ICD or ongoing ICD at CSAI onset (Supplementary Fig. 1), only three patients (8%) stopped CSAI due to ICDs (10 stopped for other reasons), whereas 25 patients (66%) continued CSAI treatment for 24 months. Of those 25 patients, six patients remained free of ICD, five maintained stable preexisting ICDs, five developed mild ICDs, one had a marked but transient ICD, and eight experienced a worsening of preexisting ICDs (Supplementary Fig. 1).

**Table 5:** Prevalence of adverse effects in percentage.

	M3	M6	M12	M24
Skin nodules	52.9	54.9	61.4	57.4
Confusion	5.8	2.8	2.9	2.9
Hallucinations	18.6	19.4	27.1	37.5
ICD^a^				
Mild	23.1	13.8	17.9	8.6
Moderate	6.2	7.7	7.5	10.3
Marked	6.2	1.5	3.0	3.4
Severe	0	0	0	0
Sedation/Drowsiness	33.3	30.6	30.0	26.5
Insomnia	14.5	18.1	21.4	11.8
Nausea	30.4	23.6	20.0	11.8
Orthostatic hypotension	23.2	22.9	24.3	31.3

Values are in percentage (%).

^a^Impulse control disorders (ICDs) such as hypersexuality, compulsive eating, compulsive shopping, pathological gambling, punding, and hobbyism. Severity of ICDs was rated according to ASBPD-4 (4 = severe disorder; 3 = marked disorder; 2 = moderate disorder; 1 = mild disorder; 0 = absence of disorder).

Other adverse effects were daytime sleepiness or drowsiness (27–33% of patients), insomnia (11–18% of patients), and confusion (3–6% of patients). Patients mean weight remained stable during the study period (68.7 (16.9) kg at M3 and 67.9 (17.4) kg at M24).

### Prediction of CSAI discontinuation

Thirty-nine patients (34%) discontinued CSAI before M24 (Fig. 1) with a mean treatment duration of 7.4 (6.4) months. Reasons for discontinuation were: drug intolerance for 16 patients (severe psychosis or hallucinations (*n* = 7), severe ICDs (pathological gambling (*n* = 2) and hypersexuality (*n* = 1)), excessive somnolence (*n* = 2), severe skin reaction (*n* = 1), troublesome nausea (*n* = 1), device intolerance (*n* = 1), cognitive deterioration (*n* = 1)), incomplete or insufficient response for 15 patients (severe OFF persistence (*n* = 10), and troublesome dyskinesias (*n* = 5)), personal decision for five patients or programmed neurostimulation surgery for three patients. Six patients who stopped CSAI because of intolerance or poor efficiency switched to DBS. When comparing the evolution during the first 6 months with CSAI of (i) the 57 patients included in the main analysis who continued CSAI during 2 years and (ii) patients who discontinued CSAI between 6 months and 24 months of follow-up (*n* = 18), there was no difference in the variation (M6–M0) of PDQ-39, motor or nonmotor status (Supplementary Table 1). The profile of antiparkinsonian drugs continuation/withdrawal was also similar (Supplementary Table 2).

The baseline characteristics (Table 1) of patients who stopped CSAI treatment (*n* = 39) were compared to patients who continued CSAI during at least 2 years (*n* = 71). They had a shorter duration of motor fluctuations (*p* = 0.018) and were more likely to have dyskinesias (*p* = 0.049). They had a trend toward a shorter disease duration (*p* = 0.060), a worse OFF-state Schwab and England (S&E) score (*p* = 0.059), and a worse score on PDQ-39 body discomfort dimension (52.2 vs 46.0, *p* = 0.072).

A forward stepwise logistic regression analysis including nine baseline factors (sex, disease duration, motor fluctuations duration, presence of dyskinesias, OFF-state S&E, Dysk UPDRS, Fluc UPDRS, ASBPD-1, and PDQ-39 body discomfort) revealed five different models for predicting treatment discontinuation (Table 6). Presence of dyskinesias (odds ratio (OR) = 4.337, *p* = 0.023), higher ASBPD-1 score (OR = 1.285, *p* = 0.016), shorter disease duration (OR = 0.901, *p* = 0.044), male sex (OR = 0.306, *p* = 0.034), and worse OFF-state S&E (OR = 0.061, *p* = 0.021) were independent predictive factors of CSAI discontinuation. The model was a significant predictor of CSAI discontinuation (*χ*^2^ = 25.23, *p* < 0.001, Nagelkerke *R*^2^ = 0.350).

**Table 6:** Stepwise logistic regression analysis for variables predicting treatment discontinuation.

	Variables	*β*	S.E.	*p* value	OR (95% CI)
Model 1	S&E, OFF	−2.496	1.028	0.015	0.082 (0.011–0.618)
Model 2	S&E, OFF	−2.36	1.057	0.026	0.094 (0.012–0.75)
	ASBPD-1	0.207	0.096	0.032	1.229 (1.018–1.484)
Model 3	S&E, OFF	−2.656	1.105	0.016	0.07 (0.008–0.613)
	ASBPD-1	0.206	0.098	0.036	1.229 (1.013–1.49)
	Sex	−1.026	0.516	0.047	0.358 (0.13–0.986)
Model 4	S&E, OFF	−2.383	1.135	0.036	0.092 (0.01–0.854)
	ASBPD-1	0.242	0.103	0.018	1.274 (1.042–1.558)
	Sex	−1.209	0.541	0.025	0.298 (0.103–0.862)
	Dyskinesias	1.28	0.613	0.037	3.597 (1.081–11.969)
Model 5	S&E, OFF	−2.79	1.207	0.021	0.061 (0.006–0.654)
	ASBPD-1	0.25	0.104	0.016	1.285 (1.048–1.575)
	Sex	−1.183	0.557	0.034	0.306 (0.103–0.913)
	Dyskinesias	1.467	0.645	0.023	4.337 (1.224–15.368)
	Disease duration	−0.105	0.052	0.044	0.901 (0.813–0.997)

*S&E* Schwab and England activities of daily living scale, *ASBPD*-1 Ardouin scale of behavior in Parkinson’s disease, part 1, general psychological state.

### Predictors of HRQoL improvement

We defined two groups of patients (Fig. 1): patients with an improvement or stabilization on PDQ-39 at M24 (*n* = 27) and patients with a less favorable evolution on CSAI (*n* = 69) including patients who stopped CSAI (*n* = 39) or who worsened on PDQ-39 at M24 (*n* = 30).

The baseline characteristics of patients who had “improved or unchanged” after 2 years of CSAI treatment compared to those patients with a less favorable evolution with CSAI were: higher OFF UPDRS-II (25 vs 20, *p* = 0.031) and worse total PDQ-39 (49 vs 40, *p* = 0.001). There were no significant differences with respect to age of PD onset, disease duration, Fluc and Dysk UPDRS, LEDD before patients received CSAI treatment, or in nonmotors symptoms (UPDRS-I, MMS, FAB, total ASBPD, or past history of ICDs).

The forward stepwise logistic regression model included eight baseline factors (OFF-state H&Y, ON-state H&Y, OFF-state UPDRS-II, UPDRS-III, ASBPD-2, ASBPD-4, total PDQ-39, number of levodopa intakes per day). Only the baseline total PDQ-39 (OR = 1.052, 95% confidence intervals (CI) 1.010–1.096, *p* = 0.015) was predictive of “improved or unchanged” HRQoL after 2 years of treatment. The model was a significant predictor of HRQoL improvement (*χ*^2^ = 6.86, *p* = 0.009, Nagelkerke *R*^2^ = 0.120). In addition, we found a significant positive correlation between baseline PDQ-39 and the improvement on PDQ-39 at M24 (*r* = 0.37, *p* = 0.005).

## Discussion

In a real-life study of 110 consecutive advanced PD patients treated with CSAI, 65% (*n* = 71) of the patients continued the treatment over the 2-year period of follow-up. Of the 57 of 71 patients with sufficient data available, we found HRQoL remained unchanged over this period. CSAI resulted in a sustained reduction of motor fluctuations, whereas the benefit on dyskinesia was transient and mild. Patients had a positive self-evaluation of CSAI effect. The treatment was well-tolerated in most patients. Thirty-four percent of patients discontinued CSAI before achieving 2 years of treatment mainly due to poor tolerance (14%) or insufficient benefit (13%). The predictors of CSAI discontinuation were a lower OFF-state S&E, the presence of dyskinesias, a shorter disease duration, a poorer psychological status (ASBPD-1), and male sex. The only baseline predictor of a positive long-term effect of CSAI on HRQoL was a worse (higher) baseline PDQ-39 score: higher scores were associated with increased improvement after 24 months. Our findings are important for clinical practice. They provide a broad overview of what can be expected in advanced PD patients treated with CSAI in a routine care setting and clues to identify patients who are more likely to benefit from this treatment.

This study has limitations related to its observational and retrospective design. Despite a larger initial population, there was a substantial amount of missing data. We excluded from the longitudinal analysis 20% of the patients due to incomplete data of PDQ-39 assessed at M0 or M24. For missing PDQ-39 data at M3, M6, and M12 (around 2%), we applied validated statistical data replacement rules (nearest available observation, NAO). Also, we did not assess nonmotor symptoms with a dedicated scale, such as the non-motor symptoms scale (NMSS). Instead, we used the UPDRS-I for this evaluation.

Real-life studies represent an important complementary approach to bridge the gap between efficacy demonstrated in RCT and effectiveness in everyday clinical practice. In routine care, we often deal with PD patients older than those included in RCT or patients who are suffering from comorbidities. We also have to consider long-term efficacy and tolerability as well as adherence rate and reasons for cessation of CSAI over time, which is rarely reported in randomized controlled studies. Our study provides important data on the effect of CSAI treatment on HRQoL in a real-life setting. One of the strengths of our work is the large sample size: only one short-term retrospective study had a similar large sample size^15^, while the two long-term retrospective studies had smaller samples^11,17^. All patients were consecutively analyzed at fixed dates for 24 months and underwent a structured assessment designed to cover all major aspects of HRQoL, motor and nonmotor symptoms. We also included a heterogeneous but realistic population of PD patients, especially regarding their age but also their neurosurgical status (patients already treated by DBS, waiting for DBS, or switching to DBS).

HRQoL remained stable over a 2-year period despite a sustained reduction in motor fluctuations. Furthermore, good treatment satisfaction was observed as assessed by a home-made satisfaction self-questionnaire. This paradox likely reflects the poor long-term effect of CSAI on nonmotor manifestations in our patients. Our nonvalidated, which had been designed for this study, questionnaire mainly focuses on motor aspects, and may have overestimated the results. External factors such as improvement on motor fluctuation could have influenced the subjective response of our patients on other aspects. By contrast, PDQ-39 is a cross-sectional quantitative scale which covers several aspects of HRQoL including physical, mental, and social domains of life. Therefore, PDQ-39 provides a better evaluation of nonmotor aspects of PD. Moreover, while some interventions have been associated with an improvement in PDQ-39^20,21^, it has been suggested that from a methodological point of view, this scale may intrinsically be more responsive to decline than improvement^22^. Indeed, nonmotor symptoms represent a major determinant of HRQoL in PD patients and there is a close association between nonmotor symptoms and HRQoL^23–25^. Mood/apathy, sleep/fatigue, and miscellaneous domains of the NMSS score were the most significant factors for variance of PDQ-39 in a multicenter cross-sectional study of 411 patients^23^. Some patients with more limited alteration of HRQoL might have also ended up disappointed due to unrealistic expectations, which could have possibly resulted in worse PDQ-39 ratings. The natural disease progression in such patients with advanced PD with the appearance of levodopa-resistant motor and nonmotor symptoms could also have influenced the observed long-term responsiveness. As a whole, the lack of HRQoL worsening over a 2-year period could be interpreted as a positive effect of CSAI in this group of patients with advanced PD. Long-term data of CSAI effect on HRQoL is scarce. Previous reported studies have suggested a short-term positive effect of CSAI on HRQoL (Table 7). Four nonrandomized, open-label studies found a significant improvement from 10 to 30% on HRQoL at 6 months^12,13,15,16^. Only two studies focused on long-term HRQoL. Rambour et al.^17^ with a follow-up of 27.9 (24.9) months did not find any difference on PDQ-39, while Martinez et al.^11^ compared CSAI vs conventional treatment over 12.5 (11.5) months and showed significant improvement on PDQ-8 within the CSAI group.

**Table 7:** Data from previous studies that evaluated the effect of CSAI on HRQoL.

Study/year, study design	*N*	Age at CSAI onset	Follow-up period (months)	PDQ applied	PDQ baseline	PDQ follow-up	Results *p* values
Naidu et al.^54^, observational	17	62 (14)	6	PDQ-8	NA	NA	Significant improvement
Martinez-Martin et al.^11^, observational	17^a^	59.5 (11.7)	12.5 (11.5)	PDQ-8	55.70	32.35	Relative change = −41.9%, *p* = 0.001
Rambour et al.^17^, retrospective	27	63.1 (9.3)	27.9 (24.9)	PDQ-39	71	77	No changes on quality of life, *p* = 0.4843
Martinez-Martin et al.^12^, prospective	43^b^	62.3 (10.6)	6	PDQ-8	49.85 (16.59)	35.03 (18.00)	Relative change = −29.75%, *p* < 0.0001
Drapier et al.^15^, prospective	100	66.7 (10.8)	6	PDQ-39	41.2 (15.5)	36.5 (13.9)	Relative change = −11.3%, *p* = 0.0114; significant improvements in the BD, stigma, mobility, and EMO
Auffret et al.^16^, observational	12	66.7 (10.8)	6	PDQ-39	33.8 (17.7)	29.6 (14.3)	Trends toward improvement in total PDQ-39 (*p* = 0.08), ADL (*p* = 0.08), and bodily discomfort (*p* = 0.09)
Katzenschlager et al.^14^, RCT	53^c^	63.6 (9.3)	12 weeks	PDQ-8	32.67	32.61	No changes on quality of life
Dafsari et al.^13^, prospective	39	62.3 (10.6)	6	PDQ-8	43.1	30.3	Relative change = −30.3%, *p* < 0.001

Values presented are mean (SD).

*BD* bodily discomfort, *ADL* activities of daily living, *EMO* emotional well-being.

^a^CSAI treated group was compared with an untreated group (*n* = 17). During follow-up, the control group showed a worsening in PDQ-8, while CSAI group showed a highly significant improvement.

^b^The study compared 43 patients on CSAI and 44 on LCIG. Difference between groups in PDQ-8 was not statistically significant.

^c^TOLEDO study. Change in PDQ-8 was not significantly different compared with placebo, mean difference −2.47 (95% CI −7.62 to 2.69, *p* = 0.3971).

Neuropsychiatric disorders, as assessed by the ASBPD, were present at baseline and did not significantly worsen within 2 years of follow-up. Sixteen patients without a history of ICDs developed ICDs. Our cohort included 38 patients with a previous history of ICDs or ongoing ICDs at CSAI initiation. Overall, these patients showed good tolerability to CSAI and most of them continued CSAI. Only four of these patients had marked but transient ICDs. Among the 39 patients who discontinued treatment, only 3 stopped due to severe ICDs.

In patients who had hallucinations or ICDs when on CSAI, our first line of action was to reduce dopamine agonist doses (oral agonists and/or CSAI). We however weighed the risk/benefit ratio between (i) reducing dopamine agonist treatments with resultant benefit on motor fluctuations and (ii) introducing clozapine to avoid dopamine agonist treatment reduction (thereby preserving motor benefits) with the risk of clozapine-induced adverse effects. Although expert consensus recommends to discontinue oral dopamine agonists in patients on apomorphine infusion^26^, in many cases, we introduced a small dose of clozapine, which reflects real-life practice in our center rather than an evidence-based approach. This allowed CSAI continuation in most patients. Our findings together with other published studies^10,11,15^ suggest an acceptable long-term behavioral tolerance profile of CSAI. Our study, as previous smaller ones, showed that CSAI seems to have low tendency to worsen or trigger ICDs^27–31^. Clinicians may thus consider the use of CSAI even in patients with a previous history of ICDs or mild ongoing ICDs, taking into account the expected risk to benefit ratio.

Cognitive status showed a significant deterioration after 12 months of treatment in our study. A previous long-term follow-up study showed that cognitive worsening tends to occur after 15 months on treatment^9^. The natural evolution of the disease likely accounts for this observation. Cognitive status was not a predictive factor of outcome in our models, possibly because the baseline cognitive status was overall relatively preserved in our patients.

PDQ-39 total score was the only baseline predictor of change in HRQoL after a 24-month treatment with CSAI in the multivariate model with a positive correlation between baseline PDQ-39 and long-term benefit on HRQoL. Patients with a poorer baseline HRQoL were more likely to improve their HRQoL. Improvement of HRQoL was independent of age, disease duration, or severity of motor complications at baseline in our study. Despite low-to-moderate dyskinesia severity at baseline in our cohort, five patients stopped CSAI because of troublesome dyskinesia. In addition, baseline dyskinesia predicted a higher risk for CSAI discontinuation and the benefit on dyskinesia seems only transient in most patients. However, the self-reported questionnaire reported a substantial improvement in dyskinesia (64%). The discrepancy between dyskinesia quantification using MDS-UPDRS and the self-reported questionnaire reveals the difficulty in assessing drug-induced dyskinesia in PD with a single scale. The multitude of dyskinesia aspects to capture (patient perceptions, duration of dyskinesia, anatomical distribution, duration, objective impairment and disability) collides with specific limitations and weaknesses of each scale^32^. MDS-UPDRS mainly focuses on dyskinesia disability and their duration and being based on just a few items, only provides a relatively limited assessment of the functional impact of dyskinesia, whereas the self-questionnaire evaluates global change. In contrast to our findings, previous studies found a clear benefit on dyskinesia when CSAI was used as monotherapy or at high flow rate with a major reduction of oral treatments^33–37^. In our real-life study, largely reducing or even stopping oral antiparkinsonian medication was not a specific goal, even in dyskinetic patients. In most patients, CSAI was rather used as an add-on treatment at relatively low doses (usual flow rate between 4–7 mg/h) in association with oral antiparkinsonian medications. It is thus likely that the oral dose reduction and apomorphine flow rate were not sufficient to induce a large improvement in dyskinesias.

Among the various subscores of PDQ-39, social support dimension deteriorated most over the 2-year period. The use of CSAI would have required increased daily support from the families and friends^4,38^. We hypothesize that some of the patients may not have received the expected support. The new constraints related to the use of CSAI and the therapeutic effect of this treatment might also have altered the dynamic of the familial functioning^39,40^. These various factors could have participated in a deterioration of the social subscore.

Altogether, our findings suggest that the best candidates for CSAI in advanced fluctuating PD are with: (i) poor baseline HRQoL and (ii) marked motor fluctuations. Adequate support from the family and friends might also be critical.

Clinicians have three device assisted therapeutic option for advanced PD patients, namely CSAI, LCIG infusion, and DBS^1^. In a real-life observational study, PD patients treated with CSAI, LCIG infusion, or bilateral subthalamic DBS, respectively, had improved PDQ-8, UPDRS-IV, and NMSS scores at 6 months^13^. By contrast to our findings, improvements in HRQoL over 36 months were found after DBS^41^ or over 24 months after LCIG infusion^42,43^. Patients included in these studies might have significantly differed from patients in our study, due to the application of more stringent eligibility criteria. Unfortunately, no study directly compares the long-term effect of all three options. Tailoring individual therapy for the individual patient is based on limited evidence regarding individualized efficacy and tolerability. In the absence of a comparative study, therapeutic choice remains challenging^35^.

## Methods

We retrospectively collected data from consecutive PD patients, who have been treated with CSAI in routine care at the Salpêtrière University hospital (Paris) over a 6-year period and described their 2-year follow-up. All patients fulfilled the Movement Disorder Society clinical diagnostic criteria for PD^44^. CSAI introduction was decided in routine care after medical evaluation and patient informed agreement.

### Standard protocol approvals and patient consents

The study protocol was approved by a local ethics committee (CPP Ile de France 6, Paris). The study was classified as an observational study (class of evidence IV) and the data collection was then approved by the national commission for data protection (CNIL) according to the French regulation rules. Patients have been informed that their medical charts could be used for research purpose after anonymization of the data. No written consent was required by the ethics committee.

### Treatments procedures

Patients started CSAI treatment during a 2-week hospital stay (baseline = M0). The initial dose was 0.5–1 mg/h and then gradually increased until the best clinical response was obtained during this hospitalization. The oral and/or transdermal antiparkinsonian treatments were gradually reduced as tolerated (prioritizing dopamine agonist’s reduction). In the absence of contraindications, domperidone treatment was prescribed in patients with digestive intolerance to apomorphine. Patients were advised how to prevent subcutaneous nodules (Supplementary Information).

Patients were readmitted for 5 days at 3 (M3), 6 (M6), 12 (M12), and 24 (M24) months after CSAI initiation to assess quality of life, efficacy, and tolerance of the treatment. During this 2-year period, the clinical teams performed therapeutic adaptations as part of routine care to optimize clinical response.

### Clinical assessments

Demographic data (age, sex), age at PD onset, disease duration, PD motor subtype (akinetic-rigid type vs tremor-dominant type), duration of motor fluctuations (period, in years, between the first appearance of motor fluctuations and the date of assessment), and presence of dyskinesias at inclusion were collected at baseline. All treatments, classified as parkinsonian, nonparkinsonian, and neurosurgical treatments, were recorded. Levodopa equivalent daily doses (LEDD) were computed^45^.

Evaluations were performed at all visits (M0, M3, M6, M12, M24). Quality of life was evaluated by PDQ-39 (range 0–100)^46^. This scale was subdivided in two subscores: physical (including mobility, activities of daily living, and bodily discomfort dimensions) and psychological (corresponding to emotional well-being, stigma, social support, cognition, and communication dimensions). Motor status was assessed according to the UPDRS-II^47^ (OFF and ON conditions, range 0–52), UPDRS-III (quantified in ON state, range 0–108), and UPDRS-IV (range 0–23); the H&Y (OFF and ON conditions, range 0–5)^48^ and the S&E activities of daily living scale (OFF and ON conditions, range 0–100%)^49^. We defined two subscores based on UPDRS-IV: Dysk UPDRS corresponding to dyskinesias assessment (items 32–35 of UPDRS-IV, range 0–13) and Fluc UPDRS corresponding to motor fluctuations assessment (items 36–39 of UPDRS-IV, range 0–7). Nonmotor and cognitive status were assessed with UPDRS part I (UPDRS-I, range 0–16), MMSE score (MMSE, range 0–30)^50^, and FAB (range 0–18)^51^. The ASBPD (range 0–84)^52^ was used to track changes in mood and behavior related to dopaminergic medication with ASBPD-1 quantifying the general psychological state, ASBPD-2 the overall functioning in apathetic mode, ASBPD-3 the nonmotor fluctuations, and ASBPD-4 the hyperdopaminergic behaviors.

At each follow-up visit (M3, M6, M12, M24), we applied a treatment satisfaction’s self-questionnaire containing five items (motor fluctuations, dyskinesias, gait disturbances, swallowing, and quality of life) rated from 0 (strong improvement) to 4 (strong worsening) and a three-item visual analog scale (range 0–10) indicating the level of pain, the device’s comfort, and general satisfaction with the CSAI, respectively.

Antiparkinsonian treatments (oral, transdermal, and CSAI treatments) and their adverse effects were collected at each visit; a numerical rating scale was used to quantify the nodule’s pain (range 0–10; with 0 indicating no pain). For patients who stopped CSAI before the 2-year follow-up visit, time and cause for discontinuation were also collected.

### Objectives

The key exploratory endpoint was to determine the change in HRQoL of PD patients, as measured by the PDQ-39 scale, at the 2-year follow-up. The secondary objectives were to evaluate the change in motor (UPDRS-II, UPDRS-III, UPDRS-IV, Dysk UPDRS, Fluc UPDRS, H&Y, S&E, treatment satisfaction’s self-questionnaire), nonmotor (UPDRS-I), and cognitive (MMSE, FAB, ASBPD) status during the 2 years CSAI treatment. Safety assessment included evaluation of adverse events and local tolerability. We then focused on the predictive factors of early CSAI dropout or HRQoL improvement.

### Statistical analysis

Data analysis was conducted with R (3.6.0) system. The normality of the distribution of the data was assessed by the Shapiro–Wilk test and the homogeneity of the variance by the Levene test. Variables that failed the Shapiro–Wilk or the Levene test were analyzed with nonparametric statistics. Parametric descriptive statistics were used to describe clinical characteristics of the cohort and to detect the presence of outliers.

For baseline data, we performed a comparative analysis between patients who discontinued the treatment and patients with 24 months of follow-up. Categorical variables were analyzed using Pearson’s *χ*2 test or Fisher’s exact test and quantitative variables with Student’s *t* test, if normally distributed, or the Mann–Whitney’s test, if they were nonparametric.

For the longitudinal analysis, quantitative missing data at M3, M6, and M12 were inputted using the NAO. NAO sets the missing data as equal to the distance-weighted time mean^53^ of the closest available data in time (backward and forward) for each patient. Then, we compared patient’s evolution at time points from M0 to M24 using a repeated measures related samples Friedman’s two-way analysis of variance by ranks test followed by Mann–Whitney post-hoc tests with a Bonferroni correction applied for multiple comparison. Normally distributed variables were analyzed by repeated measure ANOVA followed by the Bonferroni post-hoc test for multiple comparisons. Categorical variables were compared using McNemar–Bowker test of symmetry. A *p* value of less than 0.05 was defined as significant. Results will be presented as the mean (standard deviation, SD), unless otherwise stated.

In order to identify the clinical baseline predictors of treatment evolution, we performed two multiple logistic regressions (MLR) using stepwise forward elimination. A first MLR was used to estimate ORs and 95% CI for predicting treatment discontinuation and relevant clinical determinants. Patients who discontinued CSAI and patients who continued treatment during the 24-month follow-up defined the categorical dependent variable. A second MLR was used to estimate ORs and 95% CI for predicting the HRQoL improvement with CSAI therapy and relevant clinical determinants. Improved patients (patients with improved or stabilized PDQ-39 at M24 — relative change compared to baseline ≤ 0%) and patients with a less favorable evolution (patients who stopped CSAI or worsened on PDQ-39 at M24 — relative change > 0%) defined the categorical dependent variable. Variables with *p* < 0.2 in the univariate analysis were included in the multiple Wald test using stepwise forward elimination in both MLR. The relation between baseline PDQ-39 and PDQ-39 change between M0 and M24 was investigated with the Pearson method.

### Reporting summary

Further information on research design is available in the Nature Research Reporting Summary linked to this article.

## Supplementary information

Supplementary Information

Reporting Summary

## Data Availability

All data are available at the Department of Neurology of the Salpêtrière Hospital (Paris, France). The data that support the findings of this study are available from the corresponding author upon reasonable request.
