# Supplementary material for: Long-term effect of apomorphine infusion in advanced Parkinson’s disease: a real-life study
Source: NPJ Parkinsons Dis. 2021 Jun 11;7:50. doi: 10.1038/s41531-021-00194-7 (PMC8196159; doi:10.1038/s41531-021-00194-7)
Supplement: Supplementary file 1 — Supplementary Information [file 41531_2021_194_MOESM1_ESM.pdf]

SUPPLEMENTARY DATA

Supplemental Information:

Patients aged over 80 years

In our study, the two oldest patients were two women aged 80 with a 3-4 years' history of motor fluctuations without dementia, who continued CSAI over the two-year follow-up. The first patient had an improvement in motor fluctuations but a deterioration of HRQoL, likely driven by worsening of non-motor symptoms. Cognition remained stable. For the second patient, motor fluctuations and non-motor symptoms were stable at M24 with an improvement of HRQoL. MMSE deteriorated from 28 to 24. The treatment was overall well tolerated in these two patients.

Patient's education to prevent subcutaneous nodules

Patients were instructed to pay meticulous attention to hygiene, to disinfect the skin with an antiseptic at each catheter placement and to change infusion site every 12 hours. They were also recommended to massage the skin with cream (glycerol/vaseline/liquid paraffine) during ten minutes after removal of the needle and to use a Venimex (aspirant venom pump), as well as a heat cushion with cherry stones.

Supplemental Table 1: Evolution of quality of life, motor and non-motor dimensions with CSAI treatment at 6-months follow-up in patients who continued and discontinued CSAI

	**Continued CSAI (n = 57)**			**Discontinued CSAI (n = 18)**			p-value[c]
	**M0**	**M6**	**Delta**	**M0**	**M6**	**Delta**	
**Total PPQ-39**	42.3 (12.3)	42.9 (14.8)	0.6 (10.8)	46.4 (14.4)	47.1 (22.9)	0.7 (24.3)	0.447[b]
**Motor Status**							
UPDRS-II ON	7.9 (6.6)	8.6 (7.2)	0.7 (6.9)	9.9 (9.4)	10.7 (9.1)	0.8 (8.2)	0.963[b]
UPDRS-II OFF	22.2 (9.1)	20.4 (7.7)	-1.8 (7.5)	21.4 (8.3)	19.5 (10)	-1.9 (11.9)	0.973[b]
UPDRS-III ON	18.5 (11.1)	18.7 (13.4)	0.2 (12.3)	20.2 (15.1)	17.8 (11.4)	-2.4 (12.9)	0.466[a]
Dysk UPDRS	3.4 (2.7)	3.4 (3.0)	0.01 (2.5)	4.1 (2.9)	4.6 (3.6)	0.5 (2.1)	0.427[a]
Fluc UPDRS	3.7 (1.4)	2.7 (1.5)	-1 (1.8)	3.6 (1.9)	2.9 (2)	-0.7 (2.6)	0.855[b]
**Non-motor status**							
UPDRS I	3.0 (1.8)	2.5 (2.0)	-0.5 (1.8)	3.1 (1.9)	3.2 (2.2)	0.1 (1.6)	0.187[b]
MMSE	27.4 (3.1)	27.1 (3.4)	-0.2 (2.9)	26.3 (3.5)	27.1 (3.8)	0.9 (2.5)	0.319[b]
FAB	15.7 (2.6)	15.9 (2.4)	0.2 (2.4)	15.3 (2.9)	16.1 (2.3)	0.7 (1.8)	0.266[b]
ASBPD total	6.6 (4.4)	6.5 (4.1)	-0.2 (3.8)	8.3 (5.7)	8.3 (6.7)	-0.1 (4.2)	0.918[a]

Values are mean (sd). [c]Comparison of the variations (delta) at 6-months follow-up between patients who continued and discontinued CSAI. [a]Student test ; [b]Mann Whitney

Supplemental Table 2: Treatment adjustments at 6-months follow-up in patients who continued and discontinued CSAI

	Continued CSAI (n = 71)		Discontinued CSAI (n = 18)		p-value[a]
	M0	M6	M0	M6	
**L-dopa**	70 (99%)	64 (90%)	18 (100%)	14 (78%)	0.227
**Dopamine agonist**	48 (70%)	7 (10%)	10 (56%)	3 (17%)	0.654
**MAO inhibitors**	14 (20%)	11 (16%)	5 (28%)	4 (22%)	0.576
**COMT inhibitors**	34 (49%)	9 (13%)	7 (39%)	2 (11%)	0.502
**Amantadine**	26 (38%)	18 (26%)	3 (17%)	3 (17%)	0.341

Values are n (%) = number of patients receiving each antiparkinsonian drug at admission at M0 and at admission at M6. [a]Comparison of the number patients receiving the treatment at M6 between patients who continued and discontinued CSAI using Fisher's exact test.

Supplemental Figure 1: Characterization and evolution of patients with a previous history of Impulse control disorders (ICDs).

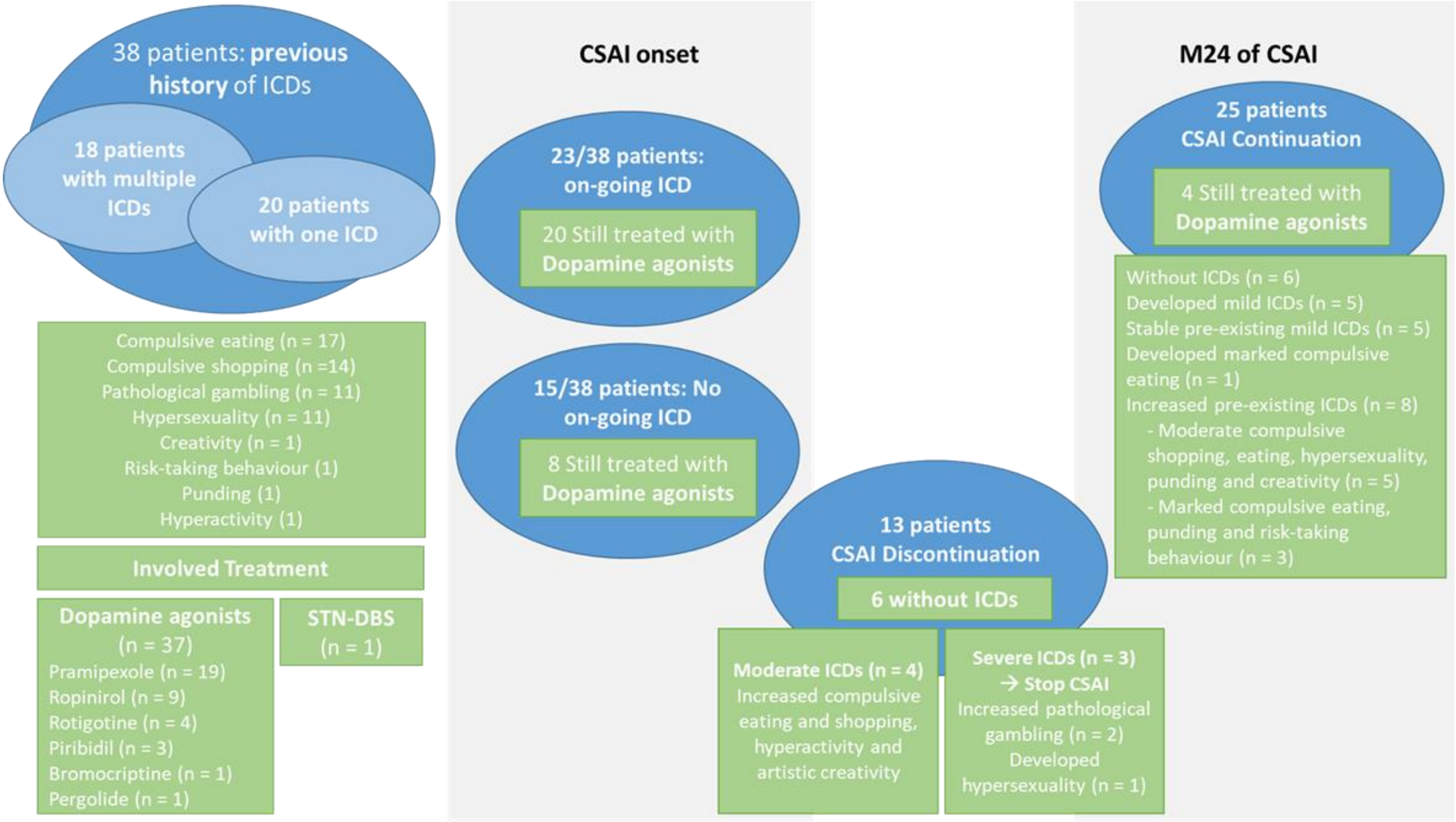

Thirty-eight patients had a history of ICDs before CSAI initiation with 18 patients having more than one type of ICDs. The most frequent ICD was compulsive eating and ICDs were mainly associated with Pramipexole. At CSAI initiation, 28 patients were still treated by dopamine agonist (mean LEDD 180,8 (161,7) mg/day) and three already received clozapine. Twenty-five patients continued CSAI at M24 and 13 discontinued treatment over the two-year period. In the group who discontinued treatment (n = 13), 6/13 patients had mild to moderate on-going ICDs at CSAI initiation (compulsive shopping, compulsive eating and punding). Three patients discontinued CSAI due to ICDs. Other 4/13 patients had a slightly worsening of pre-existing ICDs, but it was not the reason for discontinuation.

In the group who continued treatment (n = 25), 8/25 started CSAI without on-going ICDs while 14/25 had mild on-going ICDs and 3/25 had moderate ICDs (punding, hobbyism) at CSAI initiation; 19/25 were still treated with dopamine agonist and 2/25 with clozapine. Six/25 patients had a very good tolerance, with no ICDs when treated with CSAI (2 without and 4 with previous mild on-going ICDs); 5/8 without on-going ICDs at CSAI initiation developed mild non troublesome ICDs and 1/8 transiently had a marked ICD in the form of compulsive eating. Of those with mild on-going ICDs, 5/14 maintained stable ICDs and 5/14 had transient worsening to moderate ICDs. The three patients with moderate on-going ICDs had marked ICDs when on CSAI with complete resolution in one and partial resolution in two. The most frequent ICD was compulsive eating (occurring in 12 patients).

Severity of ICDs was rated according to ASBPD-4 (4 = severe disorder; 3 = marked disorder; 2 = moderate disorder; 1 = mild disorder; 0 = absence of disorder).
