# Supplementary material for: Long-term effect of apomorphine infusion in advanced Parkinson’s disease: a real-life study
Source: NPJ Parkinsons Dis. 2021 Jun 11;7:50. doi: 10.1038/s41531-021-00194-7 (PMC8196159; doi:10.1038/s41531-021-00194-7)
Supplement: Supplementary file 2 — Reporting Summary [file 41531_2021_194_MOESM2_ESM.pdf]

nature research

Corresponding author(s): DBPR NPJPARKD-00585R

Last updated by author(s): Jan 19, 2021

Reporting Summary

Nature Research wishes to improve the reproducibility of the work that we publish. This form provides structure for consistency and transparency in reporting. For further information on Nature Research policies, see our Editorial Policies and the Editorial Policy Checklist.

Statistics

For all statistical analyses, confirm that the following items are present in the figure legend, table legend, main text, or Methods section.

n/a	Confirmed	
☐	☒	The exact sample size (*n*) for each experimental group/condition, given as a discrete number and unit of measurement
☐	☒	A statement on whether measurements were taken from distinct samples or whether the same sample was measured repeatedly
☐	☒	The statistical test(s) used AND whether they are one- or two-sided<br>*Only common tests should be described solely by name; describe more complex techniques in the Methods section.*
☐	☒	A description of all covariates tested
☐	☒	A description of any assumptions or corrections, such as tests of normality and adjustment for multiple comparisons
☐	☒	A full description of the statistical parameters including central tendency (e.g. means) or other basic estimates (e.g. regression coefficient) AND variation (e.g. standard deviation) or associated estimates of uncertainty (e.g. confidence intervals)
☐	☒	For null hypothesis testing, the test statistic (e.g. *F*, *t*, *r*) with confidence intervals, effect sizes, degrees of freedom and *P* value noted<br>*Give P values as exact values whenever suitable.*
☒	☐	For Bayesian analysis, information on the choice of priors and Markov chain Monte Carlo settings
☒	☐	For hierarchical and complex designs, identification of the appropriate level for tests and full reporting of outcomes
☒	☐	Estimates of effect sizes (e.g. Cohen's *d*, Pearson's *r*), indicating how they were calculated

Our web collection on statistics for biologists contains articles on many of the points above.

Software and code

Policy information about availability of computer code

Data collection	Microsoft excel was used to collect data.
Data analysis	Data analysis was conducted with R (3.6.0) system.

For manuscripts utilizing custom algorithms or software that are central to the research but not yet described in published literature, software must be made available to editors and reviewers. We strongly encourage code deposition in a community repository (e.g. GitHub). See the Nature Research guidelines for submitting code & software for further information.

Data

Policy information about availability of data

All manuscripts must include a data availability statement. This statement should provide the following information, where applicable:

- Accession codes, unique identifiers, or web links for publicly available datasets
- A list of figures that have associated raw data
- A description of any restrictions on data availability

All data is available at the Department of Neurology of the Salpêtrière Hospital (Paris, France). The data that support the findings of this study are available from the corresponding author upon reasonable request.

Field-specific reporting

Please select the one below that is the best fit for your research. If you are not sure, read the appropriate sections before making your selection.

☒ Life sciences ☐ Behavioural & social sciences ☐ Ecological, evolutionary & environmental sciences

For a reference copy of the document with all sections, see nature.com/documents/nr-reporting-summary-flat.pdf

Life sciences study design

All studies must disclose on these points even when the disclosure is negative.

Sample size	Data was retrospectively collected from Salpêtrière University Hospital (Paris) and the sample size was the number of consecutive PD patients, who have been treated with CSAI in routine care at this hospital.
Data exclusions	116 PD patients started CSAI treatment. Six patients were excluded from the analysis (four died during the study period and two were lost to follow-up). Of the 71 patients who continued treatment for 24 months, 14 patients were excluded from the analysis due to incomplete baseline and/or 24-month PDQ-39 assessment data.
Replication	We collected all participants information, including age, sex, age at PD onset, disease duration, PD motor subtype, duration of motor fluctuations, presence of dyskinesias at inclusion, treatment and LEDD. Evaluations were performed at all visits (M0, M3, M6, M12, M24) with PPQ-39, UPDRS, H&Y, S&E, MMSE, FAB and ASBPD. At each follow-up visit, we applied a treatment satisfaction's self-questionnaire and device satisfactio's self-questionnaire. Treatment adjustments and adverse effect were collected at each visit follow-up.
Randomization	This is a retrospective single-armed observational study where randomization was not applicable.
Blinding	This is a retrospective non blinded study where blinding was not applicable.

Reporting for specific materials, systems and methods

We require information from authors about some types of materials, experimental systems and methods used in many studies. Here, indicate whether each material, system or method listed is relevant to your study. If you are not sure if a list item applies to your research, read the appropriate section before selecting a response.

Materials & experimental systems

n/a	Involved in the study	
☒	☐	Antibodies
☒	☐	Eukaryotic cell lines
☒	☐	Palaeontology and archaeology
☒	☐	Animals and other organisms
☐	☒	Human research participants
☐	☒	Clinical data
☒	☐	Dual use research of concern

Methods

n/a	Involved in the study	
☒	☐	ChIP-seq
☒	☐	Flow cytometry
☒	☐	MRI-based neuroimaging

Human research participants

Policy information about studies involving human research participants

Population characteristics	Participants are patients diagnosed with Parkinson's Disease (50% male patients, mean age 63 (10) years, mean PD duration 12 (6) years)
Recruitment	Consecutive PD patients treated with CSAI in routine care at the Salpêtrière University hospital (Paris)
Ethics oversight	The study protocol was approved by a local ethics committee (CPP Ile de France 6, Paris). The study was classified as an observational study (class of evidence IV).

Note that full information on the approval of the study protocol must also be provided in the manuscript.

Clinical data

Policy information about clinical studies

All manuscripts should comply with the ICMJE guidelines for publication of clinical research and a completed CONSORT checklist must be included with all submissions.

Clinical trial registration	N/A

Study protocol	This is not a clinical trial. Data from consecutive advanced Parkinson's disease patients treated in routine care were retrospectively collected. This standardized clinical care protocol is available at the Department of Neurology of the Salpêtrière Hospital (Paris, France).
Data collection	All data were collected from patients treated and evaluated at Salpêtrière University Hospital (Paris) from 2012 to 2020.
Outcomes	The key exploratory endpoint was to determine the change in HRQoL of PD patients, as measured by the PDQ-39 scale, at the two-year follow-up. The secondary objectives were to evaluate the change in motor (UPDRS-II, UPDRS-III, UPDRS-IV, Dysk UPDRS, Fluc UPDRS, H&Y, S&E, treatment satisfaction's self-questionnaire), non-motor (UPDRS-I) and cognitive (MMSE, FAB, ASBPD) status during the two-years CSAI treatment.
